# Genome Sequence of SARS-CoV-2 Isolate Cali-01, from Colombia, Obtained Using Oxford Nanopore MinION Sequencing

**DOI:** 10.1128/MRA.00573-20

**Published:** 2020-06-25

**Authors:** Diana Lopez-Alvarez, Beatriz Parra, Wilmer J. Cuellar

**Affiliations:** a Virology Laboratory, International Center for Tropical Agriculture (CIAT), Cali, Colombia; b Faculty of Agricultural Sciences, National University of Colombia, Palmira, Colombia; c Virology Laboratory, COVID-19 UniValle Team, Department of Microbiology, Universidad del Valle, Cali, Colombia; Queens College

## Abstract

We report the genome sequence of a severe acute respiratory syndrome coronavirus 2 (SARS-CoV-2) isolate obtained from a patient with symptoms of coronavirus disease 2019 (COVID-19) who was infected in Cali, Colombia. The patient had no recent travel record and did not require hospitalization. The virus genome was obtained using Oxford Nanopore MinION sequencing.

## ANNOUNCEMENT

Severe acute respiratory syndrome coronavirus 2 (SARS-CoV-2) (family *Coronaviridae*, genus *Betacoronavirus*) is spreading in the Americas and causing coronavirus disease 2019 (COVID-19) (1). Up to 14 May 2020, more than 13,000 cases of SARS-CoV-2 infection had been reported in Colombia, with 1,237 of those in the city of Cali. Genomic information and, above all, the development of local capacity for rapid molecular characterization of pathogens are crucial for understanding the genomic composition of the virus population and responding in a timely way to this and other emerging pathogens. Portable, next-generation sequencing technologies offer a quick approach to responding to this challenge. We report the coding-complete genome sequence of SARS-CoV-2 isolate Cali-01, obtained from a Colombian patient with no recent record of international travel.

SARS-CoV-2 isolate Cali-01 was obtained from a nasopharyngeal swab from a 45-year-old patient who presented with symptoms of COVID-19, including fever, cough, and throat congestion (2). The sample was collected in viral transport medium (VTM) following WHO protocols (3) and submitted to the Virology Laboratory of the Universidad del Valle for molecular diagnostics. Viral RNA was purified from 200 μl of VTM using a QIAamp viral RNA minikit (Qiagen), and quantitative reverse transcription-PCR was carried out by following the CDC 2019-nCoV protocol (4). For genome sequencing, the RNA sample (cycle threshold value, 16) was diluted 1:10 for cDNA synthesis using random hexamer primers and was PCR amplified using a total of 218 primer pairs covering the whole virus genome (ARTIC Network amplicon sequencing protocol v2, with primers v3) (5). Libraries were prepared from 126 ng of DNA using a genomic DNA by ligation sequencing kit (SQK-LSK109; Oxford Nanopore Technologies), and sequencing was conducted using a FLO-MIN106D (R9.5) flow cell. Base calling was performed in real time using MinKNOW v2.0 (raw reads, 103,428 bases; *N*_50_, 408 bp). The assembly was performed in two steps (using default parameters). First, for quality control and filtering of reads (fragments of 400 to 700 bp), we used the gupplyplex script of the ARTIC Network bioinformatics protocol (https://artic.network/ncov-2019/ncov2019-bioinformatics-sop.html), followed by a reference assembly with minimap2 (6) and Pilon (7), using the sequence of the Wuhan-Hu-1 isolate (GenBank accession number MN908947.3). The consensus sequence was quality checked using Qualimap v2.2.1 (8) and covered the complete reference genome (29,903 nucleotides; G+C content, 40.68%) in a contig of 102,381 reads, with an average coverage depth of 1,111×.

**FIG 1:**
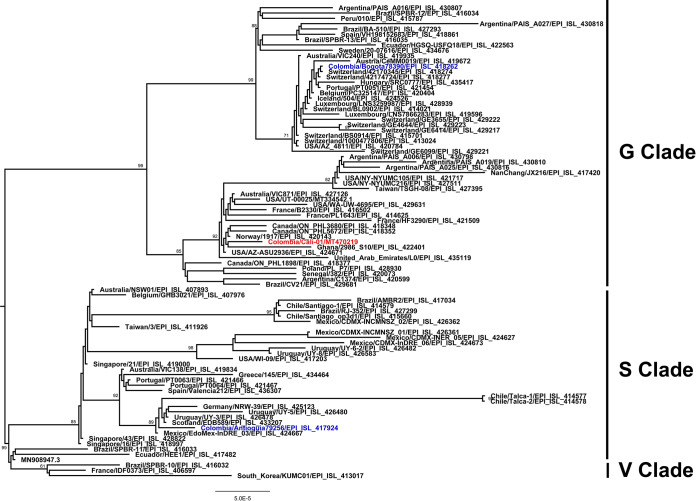
Maximum likelihood phylogeny of 89 genome sequences reported for SARS-CoV-2 isolates belonging to the G, S, and V clades, obtained from the GISAID database (https://www.gisaid.org). Values on the branches indicate bootstrap support. Isolate Cali-01 is shown in red, while other isolates from Colombia (GISAID accession numbers EPI_ISL_417924 [Antioquia] and EPI_ISL_418262 [Bogotá]) are shown in blue.

For phylogenetic analysis, a data set with 89 additional genome sequences, representing different GISAID-proposed clades (Fig. 1), was retrieved from GISAID (https://www.gisaid.org). The alignment was performed using MAFFT (9), implemented via the rapid phylodynamic alignment pipeline provided by Augur (github.com/nextstrain/augur) (10). A maximum likelihood phylogeny was reconstructed using IQ‐TREE 2.0.3 (11) with a GTR+G model, which was selected using jModelTest 2 (12). We detected seven mutations, compared to the reference genome, of which three (C241T, C3037T, and A23122T) are in a noncoding position. Mutations T265I and P4715L are located in the *orf1ab* gene, Q57H in the *orf3a* gene, and D614G in the S gene; this last change clusters isolates Cali-01 and Bogota78390 in clade G, while isolate Antioquia79256 remains in clade S (due to the L84S change in the *orf8* gene) (Fig. 1). The Cali-01 sequence reported here contributes to the ongoing international effort to track and understand the SARS-CoV-2 pandemic.

This work obtained institutional review board/ethics committee exemption from the Facultad de Salud of the Universidad del Valle because it relies on laboratory results that did not involve experimentation with humans. The required documentation is available upon request.

### Data availability.

The genome sequence of SARS-CoV-2 isolate Cali-01 was deposited in GISAID and GenBank under accession numbers EPI_ISL_445219 and MT470219, respectively. Raw reads were deposited in the GenBank Sequence Read Archive (SRA) under accession number SRX8341156 (BioProject number PRJNA632678 and BioSample number SAMN14917563).
